# Pentoxifylline Loaded Floating Microballoons: Design, Development and Characterization

**DOI:** 10.1155/2013/107291

**Published:** 2013-05-09

**Authors:** Prashant Malik, Upendra Nagaich, Raj Kaur Malik, Neha Gulati

**Affiliations:** Department of Pharmaceutics, School of Pharmacy, Bharat Institute of Technology, Meerut 250 103, India

## Abstract

The floating microballoons have been utilized to obtain prolonged and uniform release in the stomach. The objective of the present study involves design, development, and characterization of pentoxifylline loaded floating microballoons to prolong their gastric residence time. Pentoxifylline (trisubstituted xanthine derivative) loaded microballoons were prepared by the solvent evaporation technique using different concentrations of polymers like HPMC K4M and ethyl cellulose (EC) in ethyl alcohol and dichloromethane organic solvent system. Microballoons were characterized for their particle size, surface morphology, production yield, loading efficiency, buoyancy percentage, and *in vitro* drug release studies. From the characterization it was observed that increases in amount of polymers (HPMC K4M and EC) led to increased particle size, loading efficiency, and buoyancy percentage, and retarded drug release. The particle size, particle yield, loading efficiency, buoyancy percentage and *in vitro* drug release for optimized formulation (F3) were found to be 104.0 ± 2.87 µm, 80.89 ± 2.24%, 77.85 ± 0.61%, 77.52 ± 2.04%, and 82.21 ± 1.29%, respectively. The data was fitted to different kinetic models to illustrate its anomalous (non-Fickian) diffusion. The *in vitro* result showed that formulations comprised of varying concentrations of ethyl cellulose in higher proportion exhibited much retarded drug release as compared to formulations comprised of higher proportion of varying concentrations of HPMC K4M.

## 1. Introduction

Oral drug delivery systems are essential to optimize both the residence time of the system within the gastrointestinal tract and the release rate of the drug from the system. Various attempts have been made to prolong the residence time of the dosage forms within the stomach [1]. Rapid gastrointestinal transit could result in incomplete drug release from the drug delivery system to absorption window leading to diminished efficacy of the administered dose. Prolonged gastric retention is important in achieving control over the gastric residence time because it helps to maintain the controlled release system in the stomach for a longer time in an expected manner [2]. Floating systems (hydrodynamically controlled systems) are low-density systems which means they are less dense than gastric fluid. These systems have sufficient buoyancy to float over the gastric contents and remain buoyant in the stomach for a prolonged period of time without disturbing the gastric emptying rate. While the formulation is floating on the gastric contents, drug is released slowly from it at a desired rate [3, 4]. For oral sustained release, multiple unit dosage forms (i.e., microballoons) are more beneficial than single unit dosage forms. Because single unit dosage forms have the disadvantage of a release all or nothing emptying process and multiple unit dosage forms have advantages of disperse widely and release drug uniformly along the gastrointestinal tract, which results in more reproducible drug absorption, less dose dumping, and reduced risk of local irritation than the use of single unit dosage form [5–7]. Floating microballoons are gastroretentive drug delivery systems based on a noneffervescent approach. These microballoons are spherical, empty particles without core. These microballoons are characteristically free flowing powders consisting of proteins or synthetic polymers, ideally having a size less than 200 *μ*m [8]. Pentoxifylline (PTX), trisubstituted xanthine derivative (3,7-dimethyl-1-(5-oxohexyl)-3,7-dihydro-1*H*-purine-2,6-dione), is a hemorheologic agent used for the treatment of peripheral arterial disease and intermittent claudication [9]. PTX and its metabolites improve the blood flow by decreasing blood viscosity. The apparent plasma half-life of the drug and its metabolite is 2-3 hours. On the basis of using PTX as drug of choice in chronic occlusive aterial diseases, it is of a wise candidate drug to be formulated in sustained release oral dosage form [10]. Thus, the aim of the present research work was to design, develope, and characterization of pentoxifylline loaded floating microballoons.

## 2. Materials and Methods

### 2.1. Materials

Pentoxifylline was obtained as a gift sample from Bakul Pharma Pvt. Ltd. Mumbai, India. Ethyl cellulose, hydroxypropyl methylcellulose (HPMC K4M), and Tween 80 were purchased from Central Drug House (CDH), New Delhi, India. All other solvents and chemicals were of analytical grade. 

### 2.2. Methods

#### 2.2.1. Preparation of Microballoons

Microballoons were prepared by the solvent evaporation technique [11]. Pentoxifylline, HPMC K4M, and ethyl cellulose were dissolved in a mixture of ethanol and dichloromethane at room temperature. These were poured into 250 mL of water containing 0.01% Tween 80 maintained at a temperature of 30–40°C and consequently stirred at ranging agitation speed to allow the volatile solvent to evaporate. The formulated microballoons were filtered, washed with distilled water, and dried at 40°C. The composition of various formulations is shown in Table 1.

#### 2.2.2. Characterization of Microballoons

*Particle Size*.  The size of microballoons of each formulation was determined using a microscope fitted with an ocular micrometer, and stage micrometer and average particle size was determined [12]. 

*Surface Morphology*.   The surface morphology of microballoons was examined by scanning electron microscopy (Jeol JSM-1600, Tokyo, Japan) operated at 15 kV on samples gold sputtered at 10 mA, under argon at low pressure. 

*Determination of Production Yield*.   The prepared microballoons were collected and weighed. The weight of microballoons was divided by the total weight of all the nonvolatile components that were used for the preparation of the microballoons and multiplied by 100 gives the % yield of microballoons [5] as follows:

(1) % Yield=(weight  of  microballoons  collected) ×(weight of all nonvolatile  components    used  for  the  preparation)−1×100.

*Percentage Loading Efficiency*.   To determine loading efficiency, microballoons were taken, thoroughly triturated, and suspended in a minimal amount of alcohol. The suspension was suitably diluted with water and filtered to separate shell fragments. The estimation of drug was carried out using UV spectrophotometer (UV-VIS double beam spectrophotometer 2201, Systronics) at 272 nm *λ*_max_ [6]. The percentage loading efficiency was calculated as follows:

(2) Loading  efficiency  (%)  =amount  of  drug  actually  presenttheoretical  drug  load  expected×100.

*Buoyancy Percentage.*  The buoyancy test of the microballoons was carried out using USP II (paddle type) dissolution apparatus (DS 8000, LABINDIA). Dissolution test solution simulated gastric fluid (SGF) containing Tween 80 (0.02% v/v) was used as a dispersion medium to simulate gastric fluid. The microballoons were spread over the surface of the SGF, pH 1.2 (900 mL, 37 ± 0.5°C), which was agitated by a paddle rotated at 100 rpm for 12 h. After agitation for a previously determined interval, the microballoons that were floating and the ones that settled to the bottom of the flask were recovered separately [2]. After drying, the fraction of the microballoons was weighed. The % buoyancy of the microballoons was calculated by the following formula:

(3) % Buoyancy=weight  of  floating  microballoons  after  dryingweight  of  floating+settled  microballoons  after  drying ×100.

*In Vitro Drug Release Studies*.  The *in vitro* drug release from microballoons was determined using USP II dissolution apparatus. The dissolution test was performed using 0.1 N HCl (pH 1.2) as dissolution fluid (900 mL) maintained at 37 ± 0.5°C at 100 rpm. The samples (5 mL) of the solution were withdrawn from the dissolution apparatus for 12 h, and the samples were replaced with fresh dissolution medium each time to maintain the sink condition. Withdrawn samples were analyzed using UV-VIS double beam spectrophotometer at 272 nm against suitably constructed calibration curve. All measurements were carried out in triplicate, and average values were plotted [11]. 

*Statistical Analysis*.  Two-way analysis of variance (ANOVA) was applied to check significant differences in drug release from different formulations. Differences were considered to be statistically significant at *P* < 0.05 [6]. 

*Drug Release Kinetics*.  Data obtained from *in vitro *release study was fitted into various kinetic equations. The kinetic models used were zero order (cumulative percentage of drug release versus time), first order (log cumulative percentage of drug remaining versus time), the Higuchi model (cumulative percentage of drug release versus square root of time), and Korsmeyer-Peppas (log cumulative percent drug release versus log of time) [13]. Regression (*r* ^2^) values were calculated for the linear curves obtained by regression analysis. 

## 3. Results and Discussion

### 3.1. Preparation of Microballoons

Preparation of pentoxifylline loaded floating microballoons was done by using HPMC K4M and ethyl cellulose as sustained release polymers by the solvent evaporation technique. Ethanol and dichloromethane were used as solvents to keep both polymers and drug in solution. The solution was poured with the help of syringe into 250 mL water containing 0.01% Tween 80 maintained at a temperature of 30–40°C and subsequently stirred at ranging agitation speed to allow the volatile solvent to evaporate. The formulations F1, F2, and F3 were formulated by varying the concentration of ethyl cellulose, and formulations F4, F5, and F6 formulated by varying the concentration of HPMC K4M. Microballoons with higher concentration of ethyl cellulose gave much retarded drug release than higher concentration of HPMC K4M. 

### 3.2. Particle Size

From the result of this study, the average particle size of microballoons were found to be 74.63 ± 1.04, 85.18 ± 3.12, and 104.0 ± 2.87 for F1, F2, and F3 formulations and 82.96 ± 2.13, 99.32 ± 1.45, and 110.4 ± 2.94 for F4, F5, and F6 formulations, respectively. The particle size increased with increasing polymers concentration. This is due to the increase in viscosity of the solution and the decrease in stirring efficiency. Also with increasing polymer concentration, the hardening time of the microballoons was shortened. Therefore, a shorter time was provided for the breakup of droplets, and larger microballoons were formed [14]. 

### 3.3. Surface Morphology

**Figure 1:**
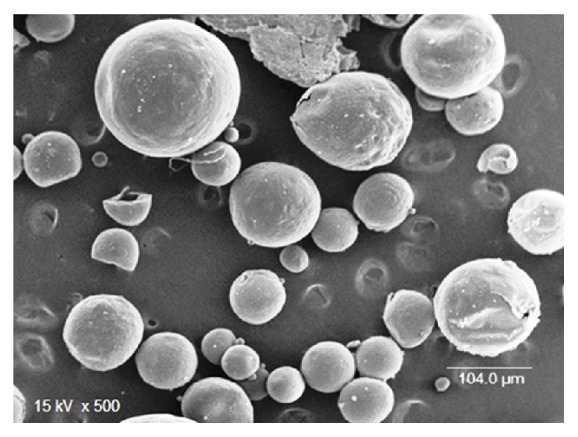
Scanning electron microphotograph of floating microballoons.

The scanning electron microphotograph showed that the developed floating microballoons were spherical with porous surface which facilitate diffusion of drug as shown in Figure 1.

### 3.4. Production Yield

Production yields were found to be 75.76 ± 1.54, 78.13 ± 1.21, 80.89 ± 2.24, 76.79 ± 1.38, 74.66 ± 2.61, and 72.57 ± 1.85 for F1, F2, F3, F4, F5, and F6 formulations, respectively, as shown in Table 2.

### 3.5. Percentage Loading Efficiency

The percentage loading efficiencies were found to be 75.5 ± 1.82, 76.36 ± 1.27,  77.85 ± 0.61, 76.22 ± 0.82, 77.29 ± 0.12, and 77.66 ± 1.35% for F1, F2, F3, F4, F5, and F6 formulations, respectively.

### 3.6. Buoyancy Percentage

The buoyancy percentage for all batches was almost above 70%, which was studied for 12 h. The highest percentage was obtained with formulation F6. Average buoyancies in percentage were found to be in the range of 72.43 ± 0.21% to 78.19 ± 0.63% for F1 to F6 formulations. In general, with the increase in the amount of polymers, there was an increase in the buoyancy percentage. The increase in the buoyancy percentage may be attributed to air and gel-forming polymer HPMC K4M which caused swelling because of increased amount of the polymers present [15].

### 3.7. *In Vitro* Drug Release Studies

**Figure 2:**
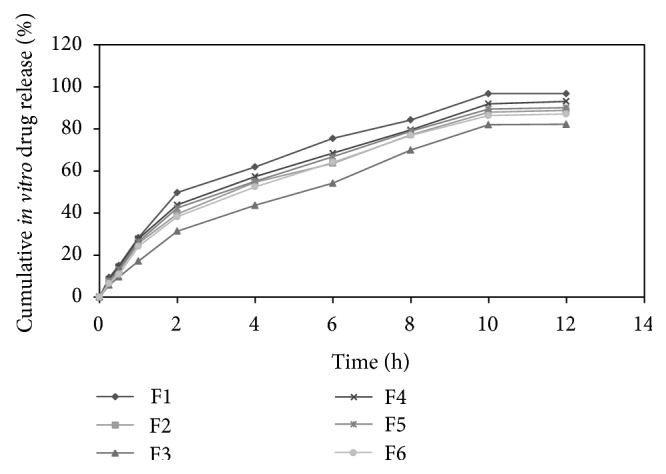
Release profile of pentoxifylline from microballoons for all formulations.

**Figure 3:**
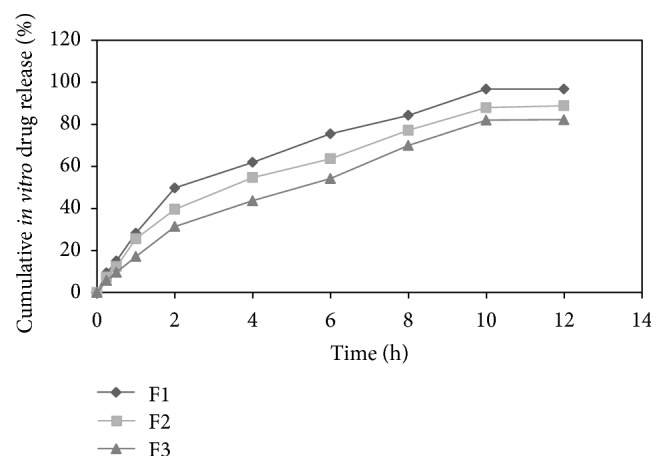
Release profile of pentoxifylline from microballoons containing varying concentrations of ethyl cellulose.

**Figure 4:**
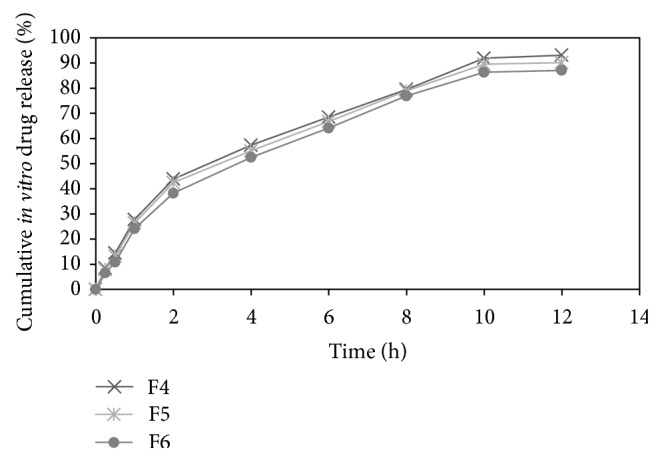
Release profile of pentoxifylline from microballoons containing varying concentrations of HPMC K4M.

The *in vitro* drug release of formulations F1, F2, F3, F4, F5, and F6 was found to be 96.81 ± 0.16, 88.84 ± 0.46, 82.21 ± 1.29, 93.13 ± 1.48, 90.16 ± 0.98, and 87.09 ± 1.73 in 12 h, respectively. Results indicate that proportion of polymers in formulation was the key factor governing the release of drug from microballoons. As the concentration of polymer increased, there was an increase in diffusional path length. This may decrease the overall drug release from the polymer matrix. Formulations comprised of ethyl cellulose in higher proportion exhibited much retarded drug release as compared to formulations comprised of HPMC K4M in higher proportion [16]. The release profile of pentoxifylline from microballoons for all formulations was shown in Figure 2. The release profile of pentoxifylline from microballoons containing varying concentrations of ethyl cellulose and HPMC K4M was shown in Figures 3 and 4, respectively.

### 3.8. Statistical Analysis

Two-way analysis of variance (ANOVA) was applied to check significant differences in drug release from different formulations containing different concentrations of HPMC K4M and ethyl cellulose. On increasing the amount of polymers, a significant decrease (*P* < 0.05) was obtained in the cumulative drug release.

### 3.9. Drug Release Kinetics

The kinetics and mechanism of drug release were determined using zero order, and first order, Higuchi's model, and further analysis was performed using Korsmeyer-Peppas equation. All formulations were found to be following Higuchi's model as the plot showed high linearity (*r* ^2^ = 0.985 to 0.991) as shown in Table 3. This equation indicates that the cumulative amount of drug release is proportional to the square root of time for diffusional release of drug from the formulation. The calculated “*n*” values from the power law equation (Korsmeyer-Peppas equation) for drug release profiles were between 0.776 and 0.842, suggesting that drug release mechanism from formulations followed the non-Fickian (anomalous) transport mechanism, which may indicate that diffusion was predominant mechanism of drug release [17]. The release kinetic data obtained from different plots of models for all formulations are given in Table 3.

## 4. Conclusion

Floating microballoons of pentoxifylline were prepared by the solvent evaporation technique using different concentrations of polymers like HPMC K4M and ethyl cellulose (EC) dispersed in ethyl alcohol and dichloromethane as a solvent system. Prepared floating microballoons showed significant floating ability, good buoyancy, and sustained drug release. *In vitro* drug release of microballoons was influenced by polymers concentration. From the percentage loading efficiency and *in vitro* drug release studies, it was observed that F3 formulation exhibits greater drug loading efficiency and sustained release behavior. On fixing the *in vitro* drug release data of optimized formulation to various kinetic models, it was found that it exhibits the Higuchi order of kinetics followed by zero order and first order. The formulation undergoes anomalous (non-Fickian) diffusion, which indicates that the drug release rate was controlled by swelling, erosion, and diffusion from microballoons. Thus, pentoxifylline loaded floating microballoons can prove to be potential pharmaceutical dosage form for prolonging the gastric retention time of dosage form.

## Figures and Tables

**Table 1:** Composition of pentoxifylline loaded microballoons.

Formulation code	HPMC K4M (mg)	Ethyl cellulose (mg)	Solvent ratio (ethanol + dichloromethane)	Tween 80 (%)	Drug (mg)
F1	200	200	1 : 1	0.01	200
F2	200	400	1 : 1	0.01	200
F3	200	600	1 : 1	0.01	200
F4	400	200	1 : 1	0.01	200
F5	600	200	1 : 1	0.01	200
F6	800	200	1 : 1	0.01	200

**Table 2:** Characterization of pentoxifylline loaded microballoons.

Parameters	Formulation code					
	F1	F2	F3	F4	F5	F6
Particle size (*μ*m)^a^	74.63 ± 1.04	85.18 ± 3.12	104.0 ± 2.87	82.96 ± 2.13	99.32 ± 1.45	110.4 ± 2.94
Production yield (%)^a^	75.76 ± 1.54	78.13 ± 1.21	80.89 ± 2.24	76.79 ± 1.38	74.66 ± 2.61	72.57 ± 1.85
Incorporation efficiency (%)^a^	75.5 ± 1.82	76.36 ± 1.27	77.85 ± 0.61	76.22 ± 0.82	77.29 ± 0.12	77.66 ± 1.35
Buoyancy (%)^a^	72.43 ± 0.21	74.28 ± 1.82	77.52 ± 2.04	73.64 ± 1.73	76.24 ± 0.82	78.19 ± 0.63
*In vitro* drug release (%)^a^	96.81 ± 0.16	88.84 ± 0.46	82.21 ± 1.29	93.13 ± 1.48	90.16 ± 0.98	87.09 ± 1.73

^a^Each value indicates the mean ± SD (*n* = 3).

**Table 3:** Release kinetic data obtained from different plots of models.

Formulation code	Zero order		First order		Higuchi		Korsmeyer-Peppas	
	*K*	*r* ^2^	*K*	*r* ^2^	*K*	*r* ^2^	*n*	*r* ^2^
F1	7.918	0.896	0.104	0.534	30.31	0.985	0.776	0.522
F2	7.341	0.922	0.106	0.569	27.79	0.991	0.799	0.565
F3	7.014	0.956	0.112	0.639	26.03	0.987	0.842	0.633
F4	7.570	0.915	0.104	0.547	28.77	0.991	0.776	0.534
F5	7.426	0.915	0.105	0.557	28.20	0.990	0.788	0.549
F6	7.296	0.925	0.109	0.586	27.56	0.989	0.825	0.592
